# Impact of obesity on critical care adult patients

**DOI:** 10.1186/cc14682

**Published:** 2015-09-28

**Authors:** Bruno F Mazza, Claudia M Antunes, Fernando Costa, Lucas J Ricotta, Luciano Quaini, Paulo F Marotto, Rosa GA Rocha, Samantha L de Almeida

**Affiliations:** 1 Hospital Samaritano, Higienópolis, São Paulo, São Paulo, Brazil

## Introduction

Obesity is a growing problem in our society today. A great number of obese patients are admitted to the ICU, and there could probably be worse outcomes and more complications during their stay in the ICU.

## Objective

The objective of this study is to analyze the epidemiological characteristics and the outcome of two groups of patients, one with a body mass index (BMI) >30 kg/m^2 ^(group 1) and another group with BMI <30 kg/m^2 ^(group 2).

## Methods

A retrospective analysis was performed from January to December 2014, using the database EPIMED^®^. We evaluated the epidemiological characteristics of patients and the outcome of them. Statistical analysis was performed using SPSS 22 software. The Mann-Whitney test was used to analyze numerical variables and the chi-square test to analyze the categorical variables. We considered data statistically significant if *p *<0.05.

## Results

We evaluated 1478 patients, 327 (22 %) in the group with BMI >30 kg/m2 (G1) and 1151 (78 %) in the group with (BMI) <30 kg/m2 (G2), who were admitted to the hospital ICU. The median of age in G1 was lower than G2 (60.4 ± 17.5 vs. 65.4 ± 20.5, *p *= 0.001). There was no difference between the groups in length of stay in the critical care unit (7.0 ± 12.0 vs. 5.8 ± 8.1), in the hospital (19.9 ± 26.7 vs. 17.7 ± 25.6) and SAPS 3 score (48.4 ± 16.8 vs. 47.5 ± 16.3) (*p *= NS). There is no difference in the mortality ratio in the ICU (group 1: 5.1 % vs. group 2: 6.8 %, *p *= NS) or in the hospital (group 1: 11.9 % vs. group 2: 10.1 %, *p *= NS).

## Conclusion

Obesity did not increase the mortality rate, or the ICU or the hospital length of stay. There was no difference in the gravity score between the groups. Current prognostic scoring systems do not include BMI, possibly underestimating the risk of death, and other quality of care indexes in obese patients. New studies could be useful to clarify how BMI affects the management of obese patients in the ICU.

